# Modification of the Spike Protein for Vaccines against Enveloped RNA Viruses

**DOI:** 10.1134/S0026893321030158

**Published:** 2021-08-26

**Authors:** A. N. Vzorov, E. I. Samokhvalov, V. V. Chebanenko, D. V. Scheblyakov, A. L. Gintsburg

**Affiliations:** 1 Gamaleya National Research Center of Epidemiology and Microbiology, Ministry of Healthcare of the Russian Federation, 123098 Moscow, Russia; 2 Biological Faculty, Moscow State University, 119234 Moscow, Russia; 3 Department of Infectiology and Virology, Sechenov First Moscow State Medical University, Ministry of Health of the Russian Federation, 123098 Moscow, Russia

**Keywords:** enveloped RNA viruses, SARS-CoV-2, HIV-1, influenza A virus, spike protein, haemagglutinin, Env, fusion mechanisms, neutralizing antibodies, vaccines

## Abstract

Most vaccines work by inducing neutralizing antibodies that target the viral envelope. Enveloped RNA viruses have evolved mechanisms for surface glycoproteins to evade host immune responses, which exhibit substantial variability, even among different strains. Natural infection and vaccines using native forms of surface proteins may induce broadly neutralizing antibodies, yet with low and ineffective levels. Class I membrane-fusion proteins of enveloped RNA viruses, HIV-1, influenza A virus, SARS-CoV-2, yield a stable conformation (so-called “pre-fusion”) in providing fusion between viral and host cell membranes. Modified viral surface proteins that are based on these features induce neutralizing antibodies with activity available against a broad spectrum of circulating strains and make it possible to overcome the difficulties associated with escape/variability of viral antigen*.*

In the twentieth century, most vaccines were designed using traditional techniques which involve pathogen isolation, pathogen inactivation, and immunization, or using viral proteins in their natural forms [1]. However, new approaches should be used to “defeat” new pathogens, mainly because of the high variability in their genomes and the conformational flexibility of their surface glycoproteins. It should be noted that vaccines containing whole inactivated virions are less safe than subunit vaccines containing individual viral components.

## SPREAD OF ENVELOPED RNA VIRUSES

Most human RNA viruses are thought to be zoonotic or of zoonotic origin [2]. HIV-1, influenza A virus, and SARS-CoV-2 are enveloped viruses with RNA genomes. Owing to their high variability, these viruses were able to cross the species barrier, enter the human population, and become adapted to humans (Table 1). The epidemic spread of new viruses is most likely due to increasing population density, growing urbanization, the development of transportation, the behavior of viruses themselves, and their adaptation to humans. The global community is now in one of the most dramatic health crises of the past decade. The emergence of the new RNA-containing enveloped SARS-CoV-2 virus, the etiological agent of COVID-19, became one of the key causes of increased mortality worldwide. The SARS-CoV-2 genome is similar to both SARS-CoV (79%) and MERS-CoV (50%), but is most closely related to the two SARS-like bat viruses bat-SL-CoVZC45 and bat-SL-CoVZXC21 (88% similarity) [3]. The new SARS-CoV-2 virus was officially categorized into the *Sarbecovirus* subgenus of the *Betacoronavirus* genus.

**Table 1.** Adaptation of the zoonotic enveloped RNA viruses, HIV, influenza A virus, and SARS-CoV-2 to the human host

Factors affecting spread of viruses in the human population	The role of the surface glycoprotein (spike)
Seasonal potential for airborne transmission [4]	Responsible for cell entry. Recognizes cell receptors
Adaptation to human cells [5]	Cell tropism alteration mechanism and modulation of virus receptor specificity [6]
Asymptomatic transmission	Accumulation of adaptive mutations [7–9]
Innate immunity [10]; preexisting immunity: cross-reactive antibodies and cross-reactive T-cell immunity [11, 12]	Ability to counteract antiviral immunity [13]
Adaptive immunity; antiviral drugs	Ability to evade the immune response and antiviral therapy; genetic variability and conformational flexibility [14]

## STRUCTURAL CHANGES OF VIRAL ENVELOPE GLYCOPROTEINS DURING ENTRY INTO CELLS

**Fig. 1.**
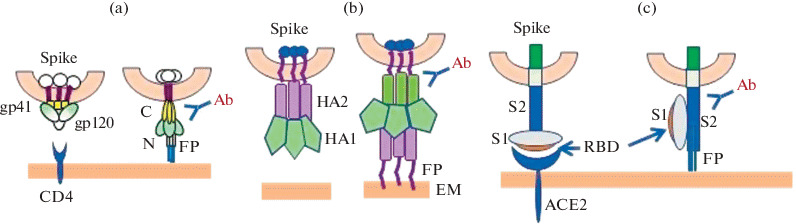
The structure of spike proteins of enveloped viruses. The spike protein exists in a metastable state on the surface of the virion: HIV (a), influenza A virus (b), and SARS-CoV-2 (c). Receptor binding (a and c),or a change in pH (b) leads to a change in protein conformation including surface subunit refolding and transmembrane subunit stabilization which results in the pre-fusion spike form. Additional epitopes inducing neutralizing antibody formation become exposed in the pre-fusion protein conformation [17–19]. The pre-fusion spike protein form may be stabilized by introducing modifications, for example, a trimerizing sequence, or by site-directed mutagenesis. FP—fusion peptide, EM—endosome membrane, RBD—receptor-binding domain, and Ab—antibody.

Envelope proteins, which have similar structural patterns in RNA viruses such as influenza A virus, SARS-CoV-2, and simian immunodeficiency virus/HIV, are the key target of the adaptive immune response. The trimeric transmembrane proteins of these viruses are Class I fusion proteins with a single transmembrane domain [15]. As part of the viral envelope, glycoproteins S (SARS-CoV), Env (HIV/SIV), and HA (influenza virus) participate in two key events: cell receptor binding, and induction of fusion of the viral and cell membranes (Table 1 and Fig. 1). Virus particles can enter the cell without binding a specific receptorvia endocytosis. However, this entry route does not result in a productive infection [16]. The fulfilment of the two conditions, the recognition of cell surface receptors as well as viral and cellular membrane fusion, results in viral entry into the cell, and triggering the virus’ replication cycle. Upon membrane fusion, viral surface glycoproteins go through a cascade of strictly regulated conformational changes resulting in stabilized protein forms.

Envelope proteins are synthesized as precursors, which are further processed to the surface and membrane-anchored subunits. The newly formed N-terminus contains the hydrophobic fusion peptide. Most Class I fusion proteins contain the receptor-binding domain (RBD), which clamps the fusion-inducing domain^1^. Protein refolding and stabilization are triggered only after virus–receptor binding when the target membrane is within reach. It is thought that it is the stabilized forms which exhibit conservative surfaces and bear the epitopes which are recognized by neutralizing antibodies with high cross-reactivity, while metastable forms have these epitopes hidden inside and expose the most variable regions which induce the production of non-neutralizing antibodies [20, 21].

## IMMUNOGENICITY OF VIRAL ENVELOPE PROTEINS

**HIV-1 envelope glycoprotein.** HIV-1 is a complex retrovirus which encodes several accessory proteins in addition to the structural proteins (Gag, Pol, and Env) present in all retroviruses. Humans possess protective immunity against simple retroviruses [22], while natural HIV infection does not induce the protective immune response. The accessory HIV-1 proteins inhibit cellular antiviral restriction factors. In addition, HIV has developed many other mechanisms that allow it to evade and even counteract the host’s protective response. One such viral factor is associated with the surface glycoprotein Env. The level of neutralizing antibodies against the Env protein produced during natural infection is insufficient to block the infection [23]. Immunogens based on native Env trimers induce a strong, although strain-specific, neutralizing antibody responses in animal models. However, immunogens based on native Env trimers are unable to create the conditions for overcoming the many obstacles on the way to the production of broadly neutralizing antibodies [24, 25]. A vaccine targeted to inhibit HIV entry into the cell will be efficient only if it induces the production of potent broadly neutralizing Env-specific antibodies.

**Influenza A virus haemagglutinin.** Haemagglutinin (HA) is the most commonly used component of anti-influenza vaccines. There exist 18 different antigenic subtypes of influenza A virus HA [26]. There are two main mechanisms which allow the influenza A virus to evade the immune response and spread in the human population, antigenic drift and antigenic shift. Antigenic drift is the result of mutations in the genes encoding the surface glycoproteins HA and neuraminidase (NA) which arise in response to the selective pressure produced by host antibodies. Antigenic shift is the emergence of new influenza A virus strains as a result of reassortment of genomic segments from different strains [27]. Current certified anti-influenza vaccines contain either inactivated, or live attenuated influenza viruses. The efficiency of protection exerted by seasonal certified vaccines changes each year depending on the antigenic correspondence between the circulating viruses and the vaccine strains and varies from 20 to 60% [28, 29]. Although influenza vaccines are effective against closely related viruses, their main limitations include the need to update the production for each epidemic season, with an uncertainty about the accuracy of the choice of current seasonal strains. When pandemic strains, which have successfully crossed the primary host–human interspecific barrier, enter the human population, vaccine development and production should be started from almost the very beginning due to the high probability of change in the influenza virus subtype.

**SARS-CoV-2 spike protein.** Coronaviruses possess genetic proofreading mechanisms [30, 31]. For this reason, the genetic divergence of SARS-CoV-2 is low compared to other RNA viruses (e.g. HIV) [32]. However, natural selection may facilitate the appearance of rare mutations favorable for the virus. Recent phylogenetic studies have demonstrated that the rate of evolution of the virus genome is accelerated in patients who suffer from long lasting infection and receive antiviral therapy, with amino acid changes occurring predominantly in the spike protein, mostly in RBD [14]. There is a study which reported protracted COVID-19 in a patient with lymphoma, which was confirmed by positive RT-PCR results for over four months. During this period the virus got 18 de novo mutations [33]. Many studies have demonstrated that the SARS-CoV-2 variant containing the D614G mutation in the spike protein rapidly became prevalent in the human population during the 2020 pandemic [34, 35]. Now, a new SARS-CoV-2 variant B.1.1.7 caused a sharp increase in the number of disease cases, first in England, and then worldwide. The B.1.1.7 genome is characterized by an unusually large number of substitutions which result in many mutations in the spike protein, three of which are the most alarming. The N501Y mutation involving one of the six key amino acid residues in RBD was identified as increasing the affinity of spike protein binding with the human and mouse SARS-CoV-2 receptor, angiotensin-converting enzyme-2 (ACE2). The 69‒70 del deletion in the S protein may have an effect on immune response evasion. The P681H mutation is in immediate proximity to the furin cleavage site, an important determinant of the virus infectious cycle [36]. Another SARS-CoV-2 variant, B.1.351, was detected in South Africa. Although B.1.351 shares some mutations with B.1.1.7 it is considered to have emerged independently from the British strain. The SARS-CoV-2 variant known as P.1 emerged in Brazil. This variant contains 17 unique mutations including 3 mutations in the RBD of the spike protein [37]. A new B.1.525 variant containing two significant mutations E484K and F888L which increase transmission and virulence of SARS-CoV-2 and attenuate the neutralizing antibody activity was registered simultaneously in Great Britain and Nigeria [38]. The analysis of the current epidemiological situation has raised the concern that even if heard immunity is obtained, SARS-CoV-2 will continue to circulate in the human population and previous infection will not protect against reinfection [39] (Table 2). For example, virus genomes isolated from a patient who had COVID-19 twice belonged to different SARS-CoV-2 strains [39]. It was reported [40] that non-neutralizing antibodies predominate in patients that have recovered from COVID-19, while the quantities of neutralizing antibodies against the SARS-CoV-2 spike protein are low (Table. 2).

**Table 2.** The comparison of the antibody response (Abs) directed against the envelope protein in HIV-1 and SARS-CoV-2 patients

Antibodies	Efficiency/level	
	HIV-1	SARS-CoV-2
Polyclonal (in blood)	No protection from reinfection	No protection from reinfection
Monoclonal (isolated from patients)	Neutralizing antibodies acting according to the effector mechanism [41]	Neutralizing antibodies acting according to the effector mechanism [42]
Neutralizing	Low level	Low level
Non-neutralizing	High level	High level

Antibodies targeted against the receptor-binding motif (RBM) of the RBD of the S protein predominate in the humoral immune response in patients who have recovered from COVID-19 [43]. Immunodominant RBM epitopes induce the production of antibodies which block S protein binding with the receptor. However, mutations in the immunodominant epitope occur much more frequently than in the non-immunodominant epitope. As a result a mutant SARS-CoV-2 may easily evade neutralizing antibodies. It has already been demonstrated that the antibody responses induced by the natural SARS-CoV-2 infection involve a wide range of S-protein epitopes (neutralizing and non-neutralizing). This phenomenon is also a notable feature of the HIV-1 surface Env protein in chronic infection when this protein exists in a relaxed conformation. In the case of the primary infection, Env exists in a compact form and mostly induces a targeted immune response directed against the neutralizing epitopes. Given this compact form, the Env can be used as a platform that produces immunogens for anti-HIV vaccines [20]. Interestingly, the affinity of monoclonal antibody binding with the RBD of the coronavirus S protein isolated from patients that recovered from COVID-19 did not correlate with neutralizing ability [44]. For example, the monoclonal 2M-10B11 antibody bound the RBD (EC_50_ 5 ng/mL) but did not neutralize authentic SARS-CoV-2; while 4А8, in contrast, showed high neutralizing activity, but did not bind RBD [44]. This may be due to the specific structure and conformational transitions in the native spike protein. On the other hand, not with standing primary RBM structures being different in SARS-CoV and SARS-CoV-2 both coronaviruses show high-affinity binding with ACE2 as with their proper receptor. Hence, the pattern of interaction between RBM and ACE2 may vary between different sarbecoviruses. However, the mechanisms of this interaction are still unclear.Fig. 1

The continuing pandemic may also facilitate the accumulation of immunologically relevant mutations in the SARS-CoV-2 genome as a result of the use of vaccines. However, it should be noted that natural SARS-CoV-2 infection may cause the production of neutralizing antibodies with a broad spectrum activity. The S309 antibodies isolated from a patient with SARS-CoV interacted with the RBD of the S glycoprotein and efficiently neutralized both SARS-CoV and SARS-CoV-2 [45]. Using cryo-electron microscopy and binding analysis S309 antibodies were found to recognize an epitope which is conserved in the *Sarbecovirus* subgenus and not compete for RBM binding with the receptor. The epitope can be reached in both the open and the closed S glycoprotein conformation [45]. It was suggested that one, or several IgG-specific bivalent mechanisms are employed in neutralization, namely, S protein trimer cross-linking (between the trimers within a single virion), creating steric constraints, or virion aggregation (as a result of virion cross-linking).

Using a primate model Yu et al. [45] demonstrated that neutralizing antibody titers induced by the anti-SARS-CoV-2 vaccine correlate with the vaccine’s protective activity.

## APPROACHES TO THE MODIFICATION OF ENVELOPE PROTEINS

To enhance the antibody response is the main goal when creating an immunogen for the antiviral vaccine. An effective immunogen may be constructed using a mechanistic strategy based on structural identification of evasion mechanisms [46]. The use of trimeric stabilized forms of viral surface proteins is drawing increasing attention in immunogen design [15]. It has already been shown that stabilized SIV/HIV Env trimers do not induce the “immune off-target responses” and non-neutralizing antibodies. The latter are formed in response to nonnative epitopes present in the natural forms of Env in viral particles [47, 48] (Table 3). The introduction of the GCN4 trimerizing sequence, the derivative of the leucine zipper motif of the yeast regulatory protein GCN4 [49], into the Env cytoplasmic domain resulted in the formation of a bundle structure stabilizing the surface subunit and exerted a significant effect on the functional activity of HIV and SIV Env proteins including modulation of the receptor-binding site exposure [50, 51]. Moreover, stabilized SIV/HIV trimers induce the production of broadly neutralizing antibodies with increased avidity, which allows them to be considered as potential immunogens for vaccines with enhanced efficiency [52].

**Table 3.** Differences between the natural and stabilized forms of the HIV/SIV, influenza A virus, and SARS-CoV-2 envelope proteins

Natural protein	Stabilized form
Metastable conformation	The pre-fusion conformation
Immune system “disorientation”	Do not cause immune system “disorientation”
Non-native epitope exposure	Exposure of the native trimer epitopes
Induction of non-neutralizing antibodies	Induction of broad-spectrum antibodies with high avidity

Regarding the influenza A virus, Weldon et al. (2010) showed that native epitopes are exposed on the soluble form of the recombinant HA trimer (sHA) containing the GCN4 trimerizing sequence at the C‑terminus, whereas unmodified sHA protein exposes the epitopes that are not exposed on the native molecule [53]. The epitopes present in the unmodified sHA are located on the “silent face” of the trimer, that is, on the monomer-monomer interface, and for this reason the antibody response is distorted. These epitopes are not exposed on the virion at physiological pH. The stabilized sHA trimer proved to be a more efficient immunogen than the unstabilized initial form and thus can be taken advantage of when developing anti-influenza vaccines. These data once again show that it is important to design an immunogen based on the structural modifications of the viral antigen.

The currently available data on the mechanism of SARS-CoV-2 entry into the host cell and antibody-mediated neutralization of this virus [42, 54, 55] allow structural design of immunogens for vaccines against highly pathogenic coronaviruses, including those which may appear in the future. For example, Rey and Lok (2018) reported that the insertion of the trimerization motif into the HIV and influenza A virus fusion proteins stabilizes the entire trimer [56]. In coronaviridae viruses, the trimerization motif did not exert any effects on the HR2 region (C-terminal heptad repeat 2) of the S protein, which existed in a disordered conformation and didn’t participate in trimer stabilization. To address this issue, Pallesen et al. (2017) introduced two proline residues (S-2P) into the HR1 of MERS-CoV, which allowed for a 50-fold increase in immunogen production in human epithelial cells compared to the native protein, the level of neutralizing antibodies induced by this protein in immunized animals was significantly higher than that obtained in the case of the unmodified molecule [57]. It has been recently demonstrated that SARS-CoV-2 S protein stabilized by the introduction of the two proline residues became conformationally homogeneous and existed in the pre-fusion conformation which displays neutralization-sensitive epitopes [55], this is importance when using S protein as an immunogen. Additional epitopes inducing the production of neutralizing antibodies become exposed in the pre-fusion conformation. A high-resolution 3D structure of the MERS-CoV S protein–G4 neutralizing antibody complex was analyzed which revealed that these antibodies are targeted against the stem region (the outer part of the fusion protein molecule) [57]. Additionally, G4-antibodies targeted against the stabilized MERS-CoV S protein form were obtained in mice. These antibodies binding with the S2 subunit are targeted against the epitopes located outside the RBD and are able to efficiently neutralize the virus [19].

The modified RNA vaccine BNT162b2 encoding the full-size SARS-CoV-2 S glycoprotein stabilized in the pre-fusion conformation induced the production of broadly neutralizing antibodies in vaccinated patients [58]. To study the efficiency of the immune response induced by the BNT162b2 vaccine, the infectious complementary SARS-CoV-2 DNA (cDNA) was obtained. Based on this cDNA Xie et al. (2021) constructed three mutant viruses with spikes composed of S protein containing the key mutations found in the recently detected British and South African variants (B.1.1.7 and B.1.351, respectively) [58]. The analysis of the sera panels obtained from 20 participants of the BNT162b2 vaccine clinical trials demonstrated that all participants produced neutralizing antibodies against all three mutant viruses at high levels. Despite the fact that blood plasma was used from those who recovered from COVID-19, the 501Y.V2 mutant may evade the neutralizing antibodies [59].

## SARS-CoV-2 VACCINES BASED ON S-PROTEINS

Currently, 48 candidate vaccines against COVID-19 are undergoing clinical trials [60]. It should be noted that S protein either as a full-size [61, 64], or as a truncated form [63] is used as an immunogen in only a few of them [61–64]. A number of modifications have also been described, including the deletion of the proteolytic cleavage site [65, 66] and the introduction of two (or more) stabilizing mutations or trimerization domains [67]. In most cases, adenoviral vectors (AdV) or recently developed RNA vaccines are used for S protein delivery and expression in cells. The published data from the preclinical studies of a number of candidate RNA vaccines make us optimistic about the future [62, 68]. However, RNA technology is a new branch of biotechnology, which means that both predicted and unpredicted setbacks may arise in large-scale vaccine production. For example, Pfizer vaccine manufacturers have already faced the problem of stability in long-term storage and the need to maintain ‒70°C. Furthermore, RNA vaccines are injectable and therefore, unlikely to cause a strong immune response in the respiratory mucosa as well as the conjunctiva, the entry gates for SARS-CoV-2.

Adenoviral vectors can be produced in large quantities and they are more stable than the RNA vaccines in that they do not require storage at low temperatures. AdV vectors efficiently stimulate both the B and T-cell immune responses, but they may be partially neutralized due to pre-existing immunity against adenoviruses. Age and pre-existing immunity against the type 5 adenovirus (AdV5) in the participants of clinical trials proved to be the two factors which affected the safety and immunogenicity of the candidate vaccine [69]. Fever was associated with younger age and a lower level of immune response to the AdV5-based vaccine delivery vector Ad5. It was found that elderly people, who were more likely to have encountered AdV5 during their lifetime, had much higher levels of neutralizing antibodies against Ad5 than young people. Therefore, elderly people may be more tolerant to a vaccine based on the Ad5-vector.

In the view of the high number of AdV5 seropositive individuals worldwide, alternative vectors are used to produce vaccines against SARS-CoV-2. Among them are AdVs belonging to the rare serotypes including types 11, 26, 35, and 49 [70], which may be used in the heterologous prime-boost schemes [61]. For example, the study of the sera samples obtained from participants involved in the clinical trials for the vaccine based on two recombinant AdV vectors rAd26 and rAd5 demonstrate that a pre-existing immunity against these two adenoviruses did not affect the antibody titers against the SARS-CoV-2 S protein RBD [61]. This may be connected to the high titers of adenovirus particles used in the analyzed vaccine, with 10^11^ per dose for each of the two recombinant viruses [61] or, the short time frame (up to 5 minutes)that it takes for AdV particles to attach themselves to the membrane and enter the cell [71].

According to the latest results of the SARS-CoV-2 vaccine trials, vaccines in which the S protein was used as an immunogen [61, 64, 72] confer up to 70 to 93% protection [73]. However, the question remains: “Which S protein form will give way to the most efficient vaccine against COVID-19, including the newly emerging SARS-CoV-2 strains?”

## CONCLUSIONS

The rapid spread of viruses may be driven by their easy host-to-host transmission, predisposition to mutational shift/drift, and neutralizing antibody evasion by new immune variants of the virus. Whether antibodies neutralize the virus or intensify the infection depends on many parameters such as specificity, concentration, affinity, and isotype [74–76]. Hypothetically, SARS-CoV-2 may be considered the result of a natural antigenic shift which occurred in SARS-CoV given the genome sequences of these two viruses is 79% identical and the repertoires of proteins encoded by them also being similar [77]. There is a certain risk that new coronavirus strains emerging as a result of antigenic drift in the circulating strains will evade the immune response induced by the parental virus or the vaccine against it, just as the influenza virus evades the antibodies induced by the seasonal vaccines [78]. This phenomenon is due to the fact that seasonal vaccines can possibly induce inefficient weakly neutralizing antibodies against the new epitopes of the mutant virus [79]. The antibody response also depends on how accessible for the antibodies the epitope on the virus particle surface is. Immunodominant epitopes on the HIV surface induce the production of non-neutralizing antibodies. This is one of the host immune system evasion mechanisms utilized by HIV. The immunodominant epitopes “divert the attention” of the B-cells from the functional trimer sites, which are not easily accessible. In the influenza A virus and SARS-CoV-2, immunodominant epitopes induce the production of antibodies which block virus entry into the cell. The SARS-CoV-2 receptor-binding motif, RBM, is the central target for the neutralizing antibodies [43]. This site is vital for the virus and its accessibility to neutralizing antibodies, playing a dual role. The epitopes of RBM are prone to natural mutagenesis, which in the long run makes it possible for the virus to evade neutralization by antibodies. It is possible, that during long-term infections this may result in antigen drift, similar to that described for the influenza A virus. It is for this reason that modified S proteins with exposed conservative epitopes (non-immunodominant) should be used to develop antibody-inducing vaccines. Notwithstanding the apparent differences in the structure and transmission routes, HIV, influenza A virus, and SARS-CoV-2 share a number of common properties. These viruses are characterized by a similar cell entry mechanism, and by the similar determinants of this process, which are surface glycoproteins recognizing their receptors on the surface of the host cell and inducing the fusion between the viral envelope and cell membrane. In HIV, influenza A virus, and SARS-CoV-2, the stabilization of the fusion protein and the exposure of its conservative surface is the key mechanism for protective antibody production [56]. New structural data might make it possible to both assess the functional importance of mutations in the S protein, which result from a genetic drift in the circulating SARS-CoV-2 strains and to match them to the known epitope regions in the stabilized S protein, which are accessible to the antibodies. This information will aid in the precise design of immunogens and accelerate the development of efficient vaccines.

## Footnotes

1 The triggers of membrane fusion (low pH or receptor binding) loosen the association between the surface and the transmembrane (TM) domains (subunits) of the spike protein thus allowing TM to get free from the clamp (certain protein fragments shield/clamp other fragments in a metastable protein form) and undergo refolding. The α-helical TM region changes its conformation and pushes another TM region, the hydrophobic fusion peptide, towards the cellular membrane.
